# Expression characteristics of the plasmid-borne *mcr-1* colistin resistance gene

**DOI:** 10.18632/oncotarget.22538

**Published:** 2017-11-20

**Authors:** Haifang Zhang, Minhui Miao, Jieting Yan, Min Wang, Yi-Wei Tang, Barry N. Kreiswirth, Xia Zhang, Liang Chen, Hong Du

**Affiliations:** ^1^ Department of Clinical Laboratory, The Second Affiliated Hospital of Soochow University, Suzhou, Jiangsu 215004, China; ^2^ Department of Laboratory Medicine, Memorial Sloan Kettering Cancer Center, Department of Pathology and Laboratory Medicine, Weill Medical College of Cornell University, New York, New York 10065, USA; ^3^ Public Health Research Institute TB Center, Rutgers University, Newark, New Jersey 07103, USA; ^4^ Department of Clinical Laboratory, The North District of Affiliated Suzhou Hospital, Nanjing Medical University, Suzhou 215008, Jiangsu Province, China

**Keywords:** mcr-1, colistin, gene expression, regulation, plasmid

## Abstract

The plasmid-encoded colistin resistance gene (*mcr-1*) has recently been reported in various Gram-negative species. However, the expression profile of *mcr-1* remains unknown. Here, we investigated the expression of *mcr-1* in various plasmids and bacteria. The *mcr-1* expression levels in pMCR1_IncX4 varied from 1.81 × 10^–5^ to 1.05 × 10^–4^ (pmol per μg total RNA) in the two *K. pneumoniae* strains SZ03 and SZ04 (ST25) and the two *E. coli* strains SZ01 and CDA6 (ST2448 and ST167, respectively). The *mcr-1* expression levels of pMCR1_IncI2 in E. coli SZ02 (ST2085) and *E. coli* BJ13 (ST457) were 5.27 × 10^–5^ and 2.58 × 10^–5^, respectively. In addition, the expression of chromosomal *mcr-1* in ST156 *E. coli* BJ10 was 5.49×10^–5^. Interestingly, after 4μg/ml colistin treatment, mcr-1 in pMCR1_IncX4 increased 2- and 4-fold at 20 and 120 mins, respectively, in all pMCR1_IncX4-harboring strains, except for ST2448 *E. coli*, which had a lower expression after 20 mins that restored to baseline levels after 120 mins. In contrast, *mcr-1* expression of pMCR1_IncI2 in the two *E. coli* strains (SZ02, BJ13) and the chromosomal *mcr-1* in *E. coli* (BJ10) remained at baseline levels after 20 and 120 mins. In the same genetic background host strain *E. coli* E600, *mcr-1* expression of pMCR1_IncX4 and pMCR1_IncI2 were similar and were decreased after colistin treatment for 20 min. However, *mcr-1* in pMCR1_IncX4 was up-regulated after colistin treatment for 120 min, while *mcr-1* in pMCR1_IncI2 was down-regulated compared to the untreated control. Our results suggested that *mcr-1* has distinct expression profiles on different plasmids, bacterial hosts, and after antibiotic treatment.

## INTRODUCTION

Multidrug-resistant Gram-negative bacteria, particularly carbapenem-resistant Enterobacteriaceae (CRE), have spread globally into hospitals and communities, and thus have become a significant public health concern [1, 2]. For clinical infections caused by CRE, the treatment options are limited and the polymixins (colistin and polymyxin B) are the last-resort antibiotic. The recent identification of a plasmid-encoded polymyxin resistance mechanism (MCR-1) in Enterobacteriaceae from both human and animal samples suggests that this last-resort antibiotic may be under jeopardy [3, 4]. To date, *mcr-1*-harboring plasmids have been identified in a number of countries with a wide geographical distribution, and some MCR-1 producing strains were resistant to multiple antibiotics [5, 6].

Polymyxin resistance is the result of a 4′-phosphoethanolamine (PEA) or 4-amino-4-deoxy-L-arabinose (L-Arap4N) modification of bacterial lipid A, which is a component of the lipopolysaccharide (LPS), and results in a reduction in polymyxin affinity. Resistance is usually chromosomally mediated and involves modulation of two-component regulatory systems (e.g., *pmrAB*, *phoPQ*, and its negative regulator *mgrB* in *Klebsiella pneumoniae*) [7, 8]. The plasmid-transferrable mobilized colistin resistance gene *mcr-1*, encoding a novel PEA-transferase [3, 4], is able to mediate a PEA addition to the lipid A moiety at the 4′-phosphate group, thereby causing colistin resistance. So far, gene expression and transcriptomic analyses of chromosomal colistin resistance genes have been reported frequently. In contrast, the number of studies on gene regulation of *mcr-1* is limited, since most of the *mcr-1* studies have been focusing on epidemiological investigations of *mcr-1* in different Enterobacteriaceae, including *Escherichia coli*, *Salmonella enterica*, *K*. *pneumonia*, *Enterobacter aerogenes* and *Enterobacter cloacae* [9–11].

Different incompatibility (InC) groups of plasmids have been found to carry *mcr-1*, while among them the IncI2 and IncX4 plasmids were most commonly reported. In the previous studies, we completely sequenced *mcr-1*-harboring plasmids pMCR1_IncX4 and pMCR1_IncI2 from clinical *K. pneumoniae* and *E. coli* strains [12]. In addition, we genomically characterized one of the first chromosomally encoded *mcr-1* genes from an *E. coli* (BJ10) isolate [13]. In this study, we used quantitative reverse transcription PCR (qRT-PCR) to evaluate the expressions of *mcr-1* of different plasmids (pMCR1_IncX4 and pMCR1_IncI2) within different species (*E. coli* and *K. pneumoniae*) and their changes in response to colistin challenge.

## RESULTS

### Analysis of the *mcr-1* locus and its promoter

**Figure 1:**
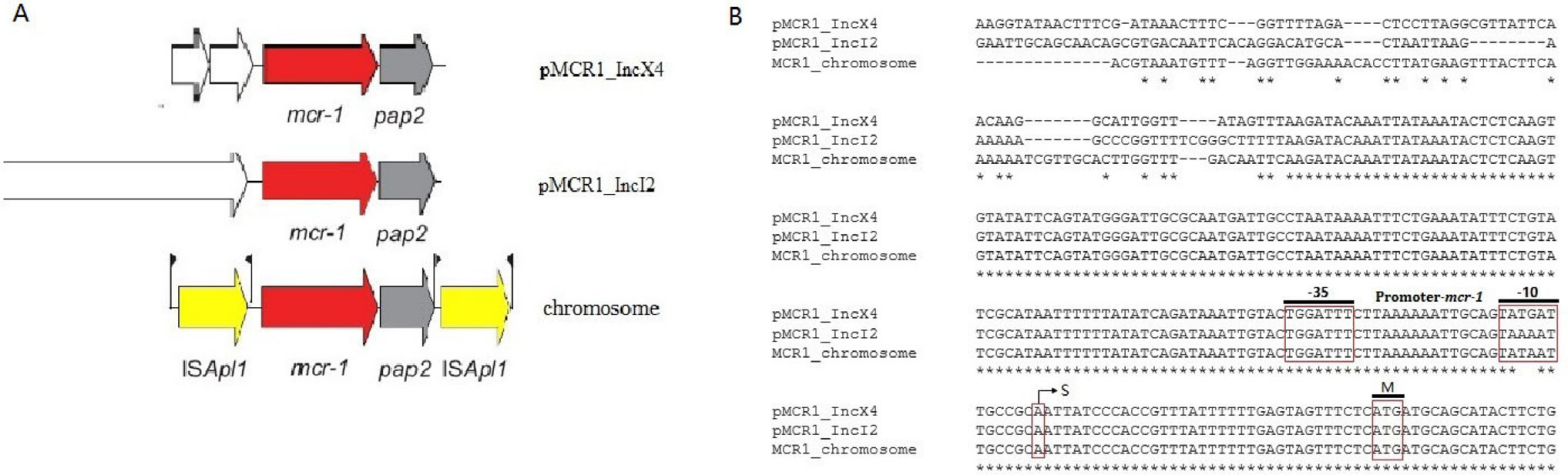
Schematic map of the different *mcr-1*- bearing plasmids pMCR1_IncX4 and pMCR1_IncI2 (**A**) Model of *mcr-1* locus. (**B**) Analyses of *mcr-1* promoter. S, transcription start site; M, methionine and translation initiation site; The *mcr-1* promoter sequences are indicated with the corresponding -10 and -35 boxes being underlined according to Poirel L’s work.

Sequence comparison of the *mcr-1* neighboring regions in pMCR1_IncX4 (accession No. KU761327), pMCR1_IncI2 (accession No. KU761326) and BJ10 (accession No. LWQZ00000000) showed that the phosphoesterase encoding gene *pap2* is universally present downstream of *mcr-1* and forms a *mcr-1-pap2* cassette, while two copies of ISA*pl1* flank the *mcr-1-pap2* cassette on the chromosome of BJ10 (Tn*Apl1*) (Figure 1A). As shown in Figure 1B, the promoter sequences of *mcr-1* in pMCR1_IncX4, pMCR1_IncI2 and BJ10 are similar to that of pAf23 and pAf48 reported by Poirel *et al.* [14].

### Expression of mcr-1 in plasmids pMCR-1_InX4 and pMCR1_IncI2 in parental strains

**Figure 2:**
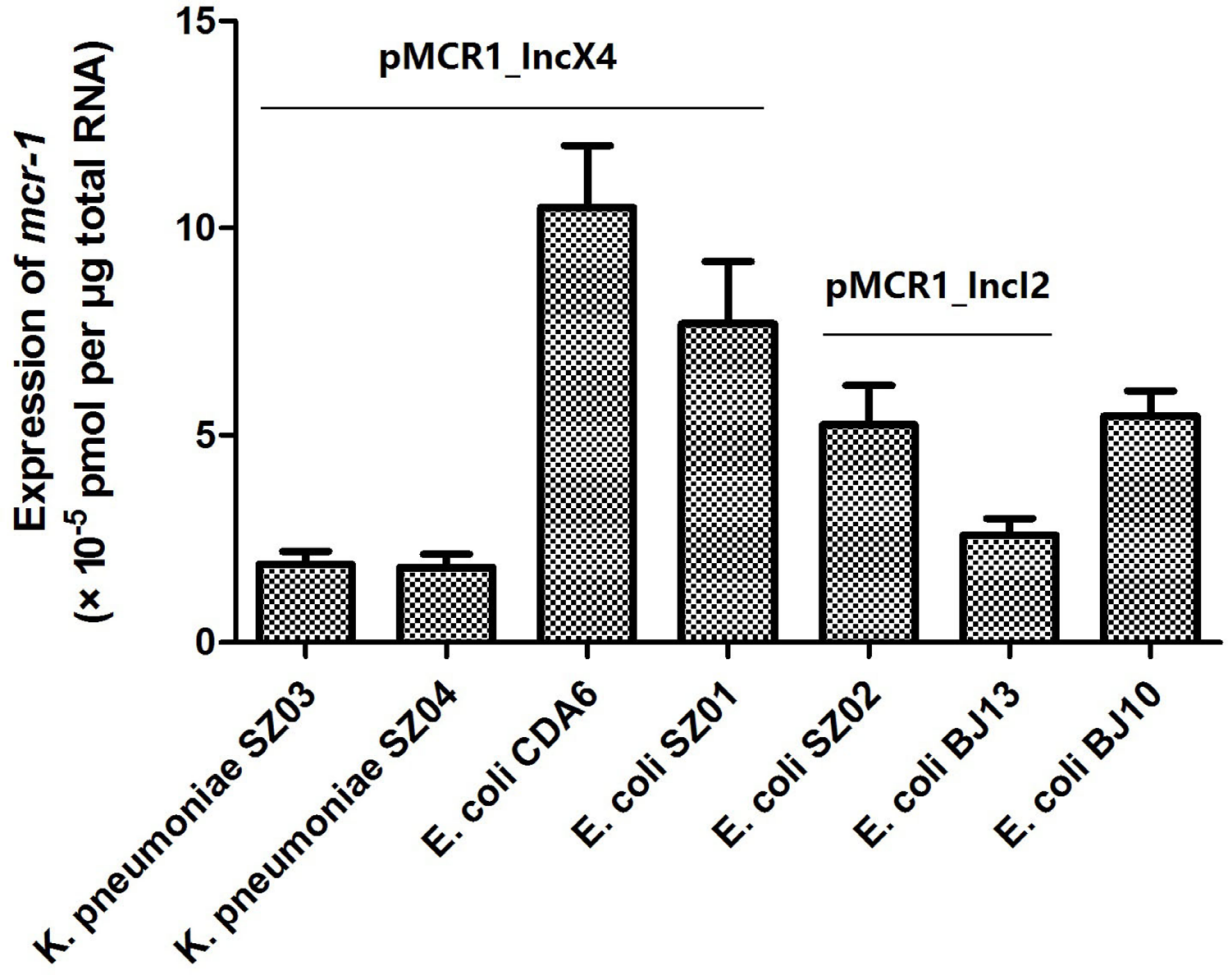
The *mcr-1* expression of plasmids pMCR1_IncX4 and pMCR1_IncI2 in parental strains Bacteria were grown to an OD_600_ of 0.5 and total RNA were extracted and subsequently used to do the qRT-PCR experiments. The data were analyzed by using Student’s *t*-test and shown as Mean with SEM.

As shown in Figure 2, *mcr-1* expressions in pMCR1_IncX4 varied from 1.81 × 10^–5^ to 1.05 × 10^–4^ (pmol per μg total RNA) in the two ST25 *K. pneumoniae* strains SZ03 and SZ04, and the two *E. coli* strains CDA6 and SZ01 (ST167 and ST2448, respectively), with the ST167 *E. coli* strain CDA6 showing the highest expression levels. The *mcr-1* expression of pMCR1_IncI2 in the ST2085 *E. coli* strain SZ02 and ST457 *E. coli* strain BJ13 was 5.27 × 10^–5^ and 2.58 × 10^–5^, respectively. In addition, the expression of chromosomal *mcr-1* (with IS*Apl1* inserted in the two flanking regions) in the ST156 *E. coli* strain BJ10 was 5.49 × 10^–5^. Our results showed that *mcr-1* expression from the same plasmid may vary significantly when expressed in the different genetic backgrounds of the different strains. In addition, we investigated the MICs for colistin in these *mcr-1* positive parental strains (Table 1) and found that the MICs for colistin were not consistent with the differences in expression of *mcr-1* in these strains. These results suggest that colistin resistance not solely depends on the expression levels of *mcr-1* and the structural modification of lipid A mediated by *mcr-1* [15].

**Table 1:** Strains and plasmids tested in this study

Strain	Background	*mcr-1*-harboring Plasmid	Colistin MIC (μg/ml)	Reference
*K. pneumoniae* SZ03	ST25	pMCR1_IncX4	32	[12]
*K. pneumoni*ae SZ04	ST25	pMCR1_IncX4	32	[12]
*E. coli* CDA6	ST167	pMCR1_IncX4	8	[13]
*E. coli* SZ01	ST2448	pMCR1_IncX4	4	[12]
*E. coli* SZ02	ST2085	pMCR1_IncI2	8	[12]
*E. coli* BJ13	ST457	pMCR1_IncI2	4	[13]
*E. coli* BJ10	ST156	$	8	[13]
*E. coli* E600	#	pMCR1_IncX4	4	This study
*E. coli* E600	#	pMCR1_IncI2	8	This study

$: *mcr-1* located in the chromosome, #: genetic engineering strain

### Expression dynamics of *mcr-1* in parental strains after colistin treatment for different times

**Figure 3:**
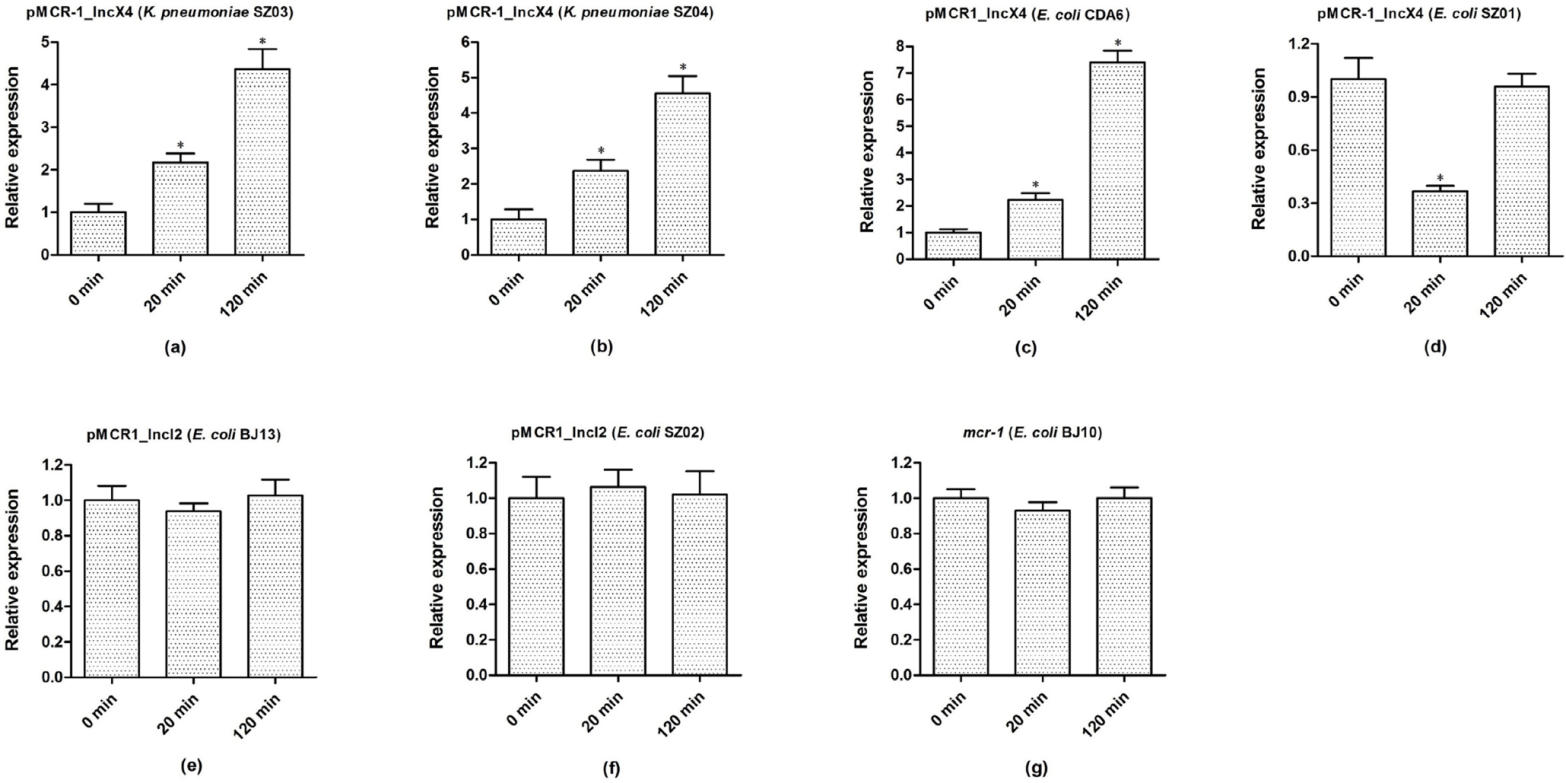
The expression dynamics of *mcr-1* in parental strains under colistin treatment for different time Bacteria were grown to an OD_600_ of 0.5, and then colistin was added into the cultures with the final concentration of 4 μg/ml. After 20 mins and 120 mins treatment by colistin, bacterial total RNA were isolated and subsequently used to do the qRT-PCR experiments. The data were analyzed by using Student’s *t*-test and shown as Mean with SEM. ^*^P < 0.05 (compared to 0 mins treatment by colistin).

We next examined *mcr-1* expression from different plasmids in different bacterial hosts after colistin treatment. As shown in Figure 3, upon colistin treatment, the expression of *mcr-1* in pMCR1_IncX4 increased 2- and 4-fold after 20 and 120 mins, respectively, among all pMCR1_IncX4-harboirng strains, except for the ST2448 *E. coli* SZ01 strain, which showed a lower expression after 20 mins that restored to baseline levels after 120 mins. In contrast, the *mcr-1* expression levels of pMCR1_IncI2-harboring parental strains SZ02 and BJ13 remained at baseline level after 20 and 120 mins. However, the expression of chromosomal *mcr-1* in *E. coli* (BJ10) remained stable, regardless of colistin treatment. These results demonstrated various *mcr-1* expression patterns of *mcr-1*-harboring plasmids in different bacterial hosts after colistin treatment, which are likely the result of differences in bacterial backgrounds and/or the different *mcr-1*-harboirng plasmids.

### Expression of *mcr-1* in *E. coli* E600 after colistin treatment for different times

**Figure 4:**
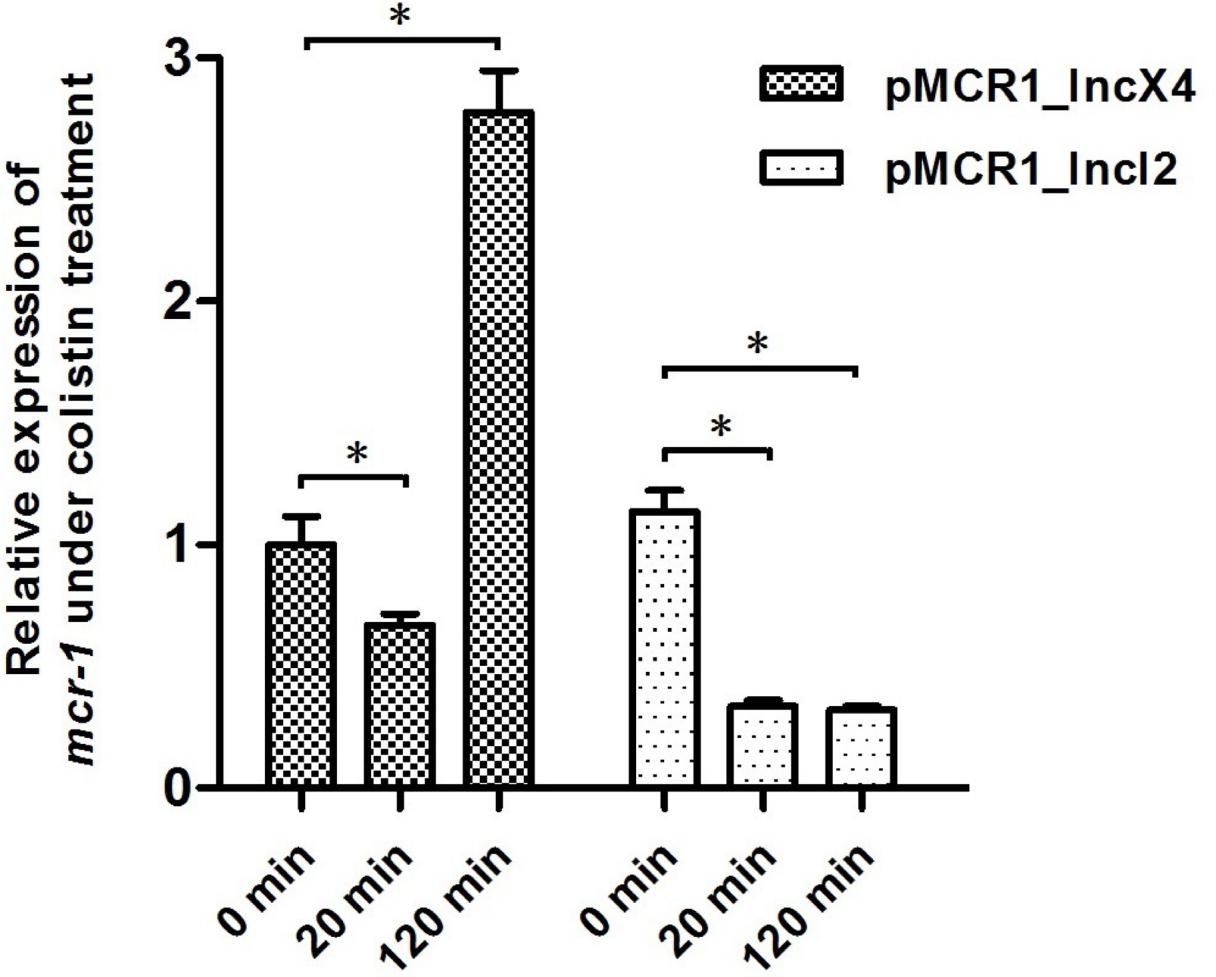
The *mcr-1* expression of plasmids pMCR1_IncX4 and pMCR1_IncI2 in same host strain *E. coli* E600 under colistin treatment for different time Bacteria were grown to an OD_600_ of 0.5, and then colistin was added into the cultures with the final concentration of 4 μg/ml. After 20 mins and 120 mins treatment by colistin, bacterial total RNA were isolated and subsequently used to do the qRT-PCR experiments. The data were analyzed by using Student’s *t*-test and shown as Mean with SEM. ^*^P < 0.05 (compared to 0 mins treatment by colistin).

To examine whether the differential expression observed above correlates with the different *mcr-1*-harboring plasmid backgrounds, we transferred plasmids pMCR1_IncX4 and pMCR1_IncI2 into the same *E. coli* host strain E600 via conjugation, and investigated the expression of *mcr-1* after colistin treatment for different times. As shown in Figure 4, the expression of *mcr-1* from the two different plasmids pMCR1_IncX4 and pMCR1_IncI2 was very similar without colistin challenge (1.31 × 10^–4^ and 1.49 × 10^–4^ pmol per μg total RNA, respectively) . However, *mcr-1* expression after colistin treatment showed variations for plasmids pMCR1_IncX4 and pMCR1_IncI2. After treatment with colistin for 20 min, *mcr-1* was significantly down-regulated in plasmid pMCR1_IncX4 and pMCR1_IncI2, while after treatment with colistin for 120 min, *mcr-1* was up-regulated in plasmid pMCR1_IncX4 but significantly down-regulated in plasmid pMCR1_IncI2.

## DISCUSSION

Colistin is a cationic polypeptide antibiotic which is regarded as one of the last antibacterial agents against CRE. In general, colistin resistance of Gram-negative bacteria like *Klebsiella*, *E. coli* and *Salmonella enterica* has been mediated by chromosomal mutations and was thought to be non-transferable. The emergence of colistin resistance mediated by the *mcr-1* gene on a plasmid has become a matter of major concern since its first report in China. To date, the *mcr-1* gene has been detected in *Enterobacteriaceae* from almost 35 countries all of the world, including *E. coli*, *E. aerogenes*, *E. cloacae*, *K. pneumonia*, *Shigella sonnei* and *S. enterica* [16–19]. A number of different plasmids including the most common, IncI2 and IncX4, have been associated with the spread of *mcr-1*. Recently, Wang *et al.* described over 10 *mcr-1*-harboring plasmids with diversified incompatibility in *E. coli* [20], and reported that the *mcr-1* promoter of different origins exhibits similar activity through transcriptional analyses [21]. However, the mechanism of *mcr-1* gene regulation is still unclear.

Previously, we identified seven CRE strains carrying the *mcr-1* gene, including two *K. pneumonia* isolates and five *E. coli* isolates, which contained the two different *mcr-1*-harboring plasmids pMCR1_IncX4 and pMCR1_IncI2 [4, 12, 13]. In this study, we found that *mcr-1* expression of the plasmids pMCR1_IncX4 and pMCR1_IncI2 may vary significantly in the different genetic background of different strains, although their promoters are highly similar. In general, gene expression is controlled by its promoter and the corresponding activators and/or inhibiters. Therefore, we suspect that genes encoding activators and/or inhibiters in the host chromosome may affect the expression of *mcr-1* located on plasmids pMCR1_IncX4 and pMCR1_IncI2. In addition, we found that the phosphoesterase encoding gene *pap2* is universally present downstream of *mcr-1* and forms the *mcr-1-pap2* cassette in pMCR1_IncX4 and pMCR1_IncI2. A previous study showed that *pap2* is likely co-mobilized with the *mcr-1* gene when it transferred from its original genetic context and does not impact colistin vsusceptibility [22].

After treatment withcolistin, various *mcr-1* expression patterns of *mcr-1*-harboring plasmids from different bacterial hosts were detected, and we suspect that the differential expression changes may be due to the differences in bacterial backgrounds and/or the different *mcr-1*-harboirng plasmids. Moreover, we found that different plasmids had similar baseline *mcr-1* expression within the same *E. coli* E600 strain, suggestin that the expression of *mcr-1* from different plasmids is likely controlled by the host genetic background without colistin challenge. However, after colistin treatment, both the host bacteria and the *mcr-1*-harboring plasmids may contribute to *mcr-1* gene expression regulation, therefore a complex regulation network of *mcr-1* may be involved.

Taken together, this is one of the first studies focusing on the expression characteristics of *mcr-1* from different plasmids and in different bacterial host backgrounds. It is suggested that different genetic signatures on the plasmids or in the bacterial strains may contribute to the observed variations, which warrant further investigations. Therefore, this study laid the foundation for further research on the regulation mechanism of *mcr-1* expression.

## MATERIALS AND METHODS

### Bacterial strains and MIC measurements of colistin

Seven *mcr-1* positive clinical isolates are used in this study, including two *K. pneumoniae* strains and five *E. coli* strains. All the bacterial strains used in this study are listed in Table 1. Minimal inhibitory concentrations (MICs) of colistin were determined using the agar dilution method and *E. coli* strain ATCC 25922 was used as a quality control strain.

### Transfer of *mcr-1* gene to the same host strain *E. coli* E600

The *mcr-1*-harboring plasmids pMCR1_IncX4 and pMCR1_IncI2 from *E. coli* SZ01 and *E. coli* SZ02, respectively, were transferred to the same host strain, *E. coli* E600, via conjugation. *E. coli* SZ01 and *E. coli* SZ02 isolates were used as donors, and *E. coli* E600 (resistant to rifampicin) was used as the recipient strain. Conjugation was carried out by broth mating and positive strains were selected by colistin and rifampicin dual resistance.

### Expression of *mcr-1* investigated by qRT-PCR

Full length *mcr-1* from pMCR1_IncX4 was cloned into a T-vector in *E. coli* DH5α, and this recombinant T-vector was used as the standard for quantitative reverse transcription PCR (qRT-PCR). The absolute expression levels of *mcr-1* of the pMCR1_IncX4 and pMCR1_IncI2 plasmids in their parental *K. pneumoniae* and *E. coli* isolates (Table 1) were investigated by qRT-PCR. In addition, the plasmid pMCR1_IncX4 and pMCR1_IncI2 were transferred into *E. coli* E600 via conjugation, and their transconjugants were evaluated by qRT-PCR. Moreover, the *mcr-1* chromosomally integrated *E. coli* (BJ10) was included in the tests [13].

In the qRT-PCR experiments, the forward primer sequence for *mcr-1* was AAATCAGCCAAACCTATCCC, and reverses primer sequence was CGTATCATAGACCGT GCCAT. The housekeeping genes *rpoD* and *gapA* were used for normalization for *E. coli* and *K. pneumoniae*, respectively. All the strains were cultured overnight at 37°C with shaking (250 rpm) in 1 ml LB broth, and then 1:100 diluted into 10 ml fresh medium. Cultures were incubated to exponential growth (OD 0.5 at 600 nm) and total RNA was extracted using an RNeasy kit (Qiagen, Germany) according to the manufacturer's instructions. Extracted RNA was treated with 1 U of RNase-free DNase I at 37°C for 10 min to remove traces of DNA and incubated at 85°C for 15 min to inactivate the DNase. Subsequently, 1 μg total RNA was subjected to qRT-PCR as described previously [23]. Each experiment was performed with three RNA samples from three independent experiments. Differences between the two groups were assessed by Student's *t*-test, *P* < 0.01 was considered to be statistically significant.

### Expression dynamics of *mcr-1* under colistin treatment investigated by qRT-PCR

To describe the expression dynamics of *mcr-1* under colistin treatment, *mcr-1* positive strains were cultured to exponential growth (OD 0.5 at 600 nm), and then colistin was added into the cultures with a final concentration of 4 μg/ml. After 20 mins and 120 mins treatment with colistin, bacterial total RNA were isolated and subsequently used to do the qRT-PCR experiments as above.
